# Gelatin-Based Films Containing Extracts of Prickly Pear (*Opuntia guerrana*): Characterization and Evaluation of Bioactive Properties

**DOI:** 10.3390/foods14223911

**Published:** 2025-11-15

**Authors:** Arely León-López, Elvia Verónica Flores-Gutiérrez, Antonio de Jesús Cenobio-Galindo, Asael Islas-Moreno, Gabriel Aguirre-Álvarez, Iván Jalil Antón Carreño-Márquez

**Affiliations:** 1 TecNM-Instituto Tecnológico Superior de Venustiano Carranza, Tecnológico S/N, Col. el Huasteco, Ciudad Lázaro Cárdenas 73049, Puebla, Mexico; arely.leon@itsvc.edu.mx; 2 Instituto de Ciencias Agropecuarias, Universidad Autónoma del Estado de Hidalgo, Av. Universidad Km 1 Rancho Universitario, Tulancingo 43600, Hidalgo, Mexico; fl468280@uaeh.edu.mx (E.V.F.-G.); antonio_cenobio@uaeh.edu.mx (A.d.J.C.-G.); asael_islas@uaeh.edu.mx (A.I.-M.); 3 Universidad Politécnica de Chihuahua, Avenida Prolongación Teófilo Borunda 13200, Labor de Terrazas, Chihuahua 31220, Chihuahua, Mexico

**Keywords:** gelatin films, *Opuntia guerrana*, biocompounds, antioxidant activity, biodegradability

## Abstract

Gelatin has been widely used as a raw material for packaging development in the food industry. Edible films made from biopolymers such as gelatin can incorporate functional ingredients from natural sources like peel powder and fresh pulp from *Opuntia guerrana* (tuna fruit). The formulations GFP, GPP, GM, and the control GF, were developed and characterized. The physicochemical composition of PP and FP (protein, fat, ash, fiber, and carbohydrates) was evaluated. Antioxidant activity showed 98.19 ± 0.21% ABTS radical inhibition for PP. FTIR analysis showed a characteristic peak at 3294–3284 cm^−1^, associated with the interaction between gelatin and hydroxyl (OH) groups from *Opuntia guerrana* phenolic compounds. The color and barrier properties of the films were affected by the addition of prickly pear peel and pulp. Mechanical properties such as Young’s modulus and tensile strength showed significant differences (*p* ≤ 0.05) when pulp was added to the films. The film with PP exhibited the highest concentration of bioactive compounds (phenols, flavonoids, and betalains) and inhibited the ABTS radical 98.24 ± 0.08% and 38.50 ± 2.11% DPPH radical. All films reached biodegradation levels of approximately 90% after 15 days of incubation. The use of prickly pear residues to obtain value-added compounds can significantly modify the physicochemical and functional properties of gelatin films.

## 1. Introduction

Food packaging plays a crucial role in the food industry, as its main objective is to protect food products from external agents. There is a wide variety of packaging sizes, colors, and shapes, as their characteristics depend on the specific food product to be protected. However, most food packages are made of synthetic polymers, and approximately 367 million tons of plastic packaging materials are produced annually, representing a major source of pollution [1]. To mitigate pollution problems associated with conventional food packaging, this study focuses on developing alternative edible films and coatings. Edible films and coatings are thin layers (<0.3 mm) that form protective coatings on foods and, in some cases, can be consumed along with the product. This type of packaging plays an important role in conservation, distribution, and marketing in the food industry. The difference between edible films and coatings is that the former are prepared separately and later applied to food surfaces, whereas the latter form directly on the product. Edible films are formed from combinations of biopolymers, plasticizers, and additives that can incorporate bioactive compounds from natural sources, improving their physicochemical and functional properties [2]. Gelatin, obtained by the partial hydrolysis of collagen, is one of the most widely used biopolymers for the preparation of edible films. It is fully biodegradable, non-toxic, biocompatible, and serves as a vehicle for antioxidant, antimicrobial, and other functional ingredients derived from vegetables and fruits such as prickly pear [3]. Prickly pear (*Opuntia* spp.) is classified as a fleshy, oval-shaped berry containing seeds and a semi-hard shell. It is a fruit native to Mexico, with approximately 400 recognized species [4,5]. Prickly pear is a rich source of vitamins; minerals (K, Mg, Ca, Na); fiber; amino acids (arginine, glutamic acid, proline); carotenoids (β-carotene, α-cryptoxanthin, lutein, zeaxanthin, spiroloxanthin, echinenone, antheraxanthin); betalains (betacyanins and betaxanthins); and polyphenolic compounds (kaempferol, quercetin, and isorhamnetin) [3,6].

Several studies have reported that the incorporation of functional compounds from various fruit sources into gelatin films or coatings improves their physicochemical and antioxidant properties [7,8]. Also, gelatin films with bioactive compounds offer the opportunity to release bioactive compounds that migrate toward the food, maintaining their quality and sensorial properties [9,10].

The main objective of this project was to elaborate edible films from gelatin with bioactive compounds from prickly pear and evaluate their properties. Therefore, this study contributes to the sustainable use of regional Opuntia resources by valorizing agroindustrial residues through the development of biodegradable food packaging materials.

## 2. Materials and Methods

The prickly pear variety used was *Opuntia guerrana*, collected between July and September in Hidalgo, Mexico. Prickly pears were washed and sanitized (100 ppm NaClO) for 5 min; afterwards, the peel and pulp were separated. The peel was cut into 1 cm^2^ pieces, dried in a convection oven (Felisa FE-293A, Zapopan, Jalisco, Mexico) at 60 °C for 48 h, milled (Nutribullet NBR-0601 WM, Los Angeles, CA, USA), and the resulting powder was stored in complete darkness at −10 °C until use. The fresh pulp was manually separated from the seeds using a sieve and stored under the same conditions as the powdered peel [11].

### 2.1. Physicochemical Analysis of PP and FP

Physicochemical analyses were conducted following AOAC methods [12]; moisture (925.09), protein (Kjeldahl method, 955.04) with a N conversion factor of 6.25, fat (Soxhlet, 920.39), ash (923.03), and fiber (962.09).

### 2.2. Extracts Obtained from PP and FP

The extracts were obtained following the method of Pinedo-Espinoza [13]. A measurement of 0.5 g of PP or FP was mixed with 20 mL of an ethanol:water (50:50, *v*/*v*) solution and centrifuged at 11,000× *g* for 20 min at 4 °C (Thermo Scientific Mod. ST 16R, Dreieich, Germany). The supernatant was the extract and was used for the antioxidant determination assay.

### 2.3. Antioxidant Activity of PP and FP

Total phenolic content was determined following the method of Espino-Manzano et al. [14]. A measurement of 0.5 g of sample was mixed with 20 mL of ethanol:water (50:50, *v*/*v*), and 0.5 mL of the mixture was placed in an ultrasonic bath (Ultrasonic Cleaner, Mod. 32V118A, Freeport, IL, USA) at a frequency of 40 kHz for 30 min at 20 °C and subsequently centrifuged at 11,000× *g* for 20 min at 4 °C (Thermo Scientific Mod. ST 16R, Germany). A measurement of 1 mL of the supernatant was mixed with 5 mL of Folin–Ciocalteu reagent diluted in water 1:10 (*v*/*v*), and after 7 min, 4 mL of the sodium carbonate reagent (7.5%) was added, allowing the mixture to react for 2 h in total darkness at room temperature. The absorbance was measured at 760 nm in a spectrophotometer (JENWAY, Model 6705, Dunmow, UK). A calibration curve of gallic acid was used as a standard. Results were expressed as mg GAE/g of sample.

Flavonoid content was determined according to the method described by Arvouet-Grand et al. [15]. A measurement of 0.1 g of sample was mixed with 10 mL of ethanol:water (50:50) for 10 min. After, 2 mL of it was mixed with 2 mL of a methanolic solution of AlCl_3_ (2%) for 20 min in total darkness. The absorbance was measured at 415 nm using a spectrophotometer. A quercetin calibration curve was used as the standard; the results were expressed as mg EQ/100 g of sample.

The betalain content was determined according to González-Aguayo [16]. One gram of sample was mixed with 10 mL of an ethanol:water (50:50, *v*/*v*) solution, vortexed for 30 min, and centrifuged at 11,000× *g* for 20 min at 4 °C. A measurement of 1 mL of the supernatant was taken and mixed with 20 mL of 20% ethanol. The absorbance was measured at 538 nm (Betacyanin) and 483 nm (Betaxanthins) according to the following equation:

BC (mg/g) = A(DF)(MW)VD/ℇLWd

BC: Betalain content;

A: 536 nm for betacyanin;

A: 483 nm for betaxanthin;

DF: Dilution factor;

MW: Molecular weight of betacyanin 550 g/mol;

MW: Molecular weight of betaxanthins 308 g/mol;

VD: Volume of dry sample in solution;

ℇ: 60,000 L/(mol cm) (Betacyanin);

ℇ: 48,000 L/(mol cm) (Betaxanthin);

L: Cell size 1 cm;

Wd: Weight of dry sample.

DPPH (2,2-diphenyl-1-picrylhydrazyl) radical inhibition activity was determined according to Brand-Williams [17]. A measurement of 300 µL of the sample was mixed with 2.7 mL of DPPH solution (6.1 × 10^−5^ M) (Sigma-Aldrich; St. Louis, MI, USA). The mixture was stirred for 15 s, leaving it to stand in total darkness for 1 h at room temperature and then read in a spectrophotometer at 515 nm.

The ABTS (2,2′-azino-bis(3-ethylbenzothiazoline-6-sulfonic acid) radical scavenging capacity was determined according to the method reported by Re [18]. ABTS solution (7 mM) was mixed with an aqueous solution of K_2_S_2_O_8_ (2.45 mM) in a 1:1 (*v*:*v*) rate. After stirring for 12–16 h in total darkness at room temperature, the ABTS solution was diluted with ethanol until an absorbance of 0.700 ± 0.02 at 754 nm was obtained. A measurement of 200 µL of the extract and 2 mL of the ABTS solution were mixed and stirred for 6 min in total darkness. The absorbance was measured at 754 nm. The results were expressed as percentage of inhibition of ABTS and DPPH radicals according to the following equation:

AA(%) = 1 − ((Sa))/((Ra)) × 100

AA: Antioxidant activity expressed as percentage of scavenging radical capacity;

Sa: Sample absorbance;

Ra: Radical only absorbance (0.700 ± 0.02 nm).

### 2.4. Edible Films Elaboration

Films were prepared using the casting method. Gelatin (2%, 275° Bloom; Merck, Darmstadt, Germany) was mixed with 150 mL of distilled water and heated (60 ± 5 °C) under constant stirring until complete dissolution; glycerol, PP, and PF were added to the solution to obtain different formulations (Table 1) and the solution of gelatin with prickly pear compounds was transferred into Petri dishes (14 cm diameter) and dried in an oven (Felisa FE-361, Mexico) at 25 °C for 48 h to obtain the films. The dried films were removed from Petri dishes and stored in polyethylene bags in total darkness until characterization [14].

### 2.5. Gelatin Edible Film Characterization

Films were evaluated for FTIR, thickness, color, and opacity after they were removed to Petri dishes, to avoid the relative humidity affecting them.

Thickness was measured using a digital micrometer (Mitutoyo, Tester Sangyo Co. Ltd., Tokyo, Japan) at eight different points on the film surface [19].

Color parameters (L, a*, b*) were determined according to the CIE system: L (lightness), a* (green to red), and b* (blue to yellow). Five different points on the film surface were selected for the measurement [20].

The opacity was evaluated following Maryam-Adilah [21], where the films were cut and put in an empty cell at 600 nm in a UV-Vis spectrophotometer (JENWAY, Model 6705, Dunmow, UK), and the opacity was calculated according to the following equation:

Opacity = Absorbance value obtained/T Thickness of the film (mm)

### 2.6. Mechanical Properties

Tensile strength (TS), Young’s modulus (E), and elongation at break (%E) were evaluated. Films were cut into strips (10 cm long × 1 cm wide) and kept in storage at 57% humidity for 7 days. A texturometer (TA-X-T PLUS, Stable MicroSystems, Godalming, UK) was used with an initial grip separation set at 50 mm and a crosshead speed at 0.10 mm/s [22].

Moisture content was determined from the weight loss of film strips (1 × 4 cm) after 24 h in a stove drying at 105 °C, according to Li et al. (2014) [23]. The final data were calculated by the following equation:

Moisture (%) = 100 × (W1 − W2)/W1

W_1_ is the initial weight of films;

W_2_ is the final weight of films after 24 h in stove.

For film solubility determination, films were cut into strips of 2 × 3 cm and put in a stove (105 °C, 24 h). Afterwards, samples were weighed and put in a glass with 30 mL of distilled water at 25 °C and stirred for 24 h; after, the solution was filtered and weighed and dried at 105 °C for 24 h in a stove [24]. Following the formula, the solubility percentage was calculated:

Solubility (%) = [(Initial dry weight − Final dry weight)/Initial dry weight] × 100

For the determination of the swelling index, film samples (2 × 2 cm) were dried in a desiccator with silica gel for 7 days and then immersed in 30 mL of distilled water for 2 min at 25 °C. Excess water was dried with filter paper, and after the sample was weighed [25]. The swelling index was calculated by the following equation:

W(%) = 100 (W_w_ − W_d_)/W_d_

W_w_: wet weight;

W_d_: dry weight.

For water vapor permeability (WVP), the “test cell” method according to Mora-Palma [26] was used. The samples were kept for 48 h at 57% humidity. After the thickness was evaluated with a digital micrometer at five different points, the films, which were previously cut into circles of 7.5 cm in diameter, were carefully placed inside the cell with silica to achieve 0% humidity. The test cells with the samples were placed in a desiccator with 75% relative humidity, and film-based gelatin weight change was evaluated every hour for 8 h. The WVP was calculated as follows:

WVP = (w × x)/(t × A × ΔP) × 100

w: Weight gained of sample (kg);

x: Thickness of films (mm);

t: Time of the experiment for the weight gain of the sample;

A: Area of film sample (m^2^);

ΔP: Difference in partial vapor pressure between the dry atmosphere and pure water.

### 2.7. Fourier Transform Infrared Spectroscopy (FTIR)

FTIR spectra of gelatin films were obtained using a Fourier transform infrared spectrophotometer (PerkinElmer, Waltham, MA, USA) equipped with an attenuated total reflection accessory. Samples were placed on the ATR diamond crystal, and spectra were recorded in absorbance mode from 4000 to 400 cm^−1^ with a resolution of 4 cm, combining 32 scans. The air spectrum was subtracted from all spectra. Bands were analyzed using the Spectrum 10.4.2 software [27].

### 2.8. Differential Scanning Calorimetry (DSC)

The thermal stability of the films was analyzed using the Q2000 series DSC equipment (TA Instruments, New Castle, DE, USA) equipped with a cooling system and TA 2000 universal analysis software v4.5a. The samples (1.0 ± 0.1 mg) were packed in hermetically sealed aluminum pans and scanned in a range of 25 to 200 °C with a heating rate of 10 °C/min, with nitrogen flow (50 mL/min). The enthalpy of denaturation (ΔHd) was calculated as the area of the endothermic denaturation peak in the first heating scan [14].

### 2.9. Biodegradability

For biodegradability assessment, gelatin films were weighed, placed on iron gauzes, and buried 2 cm deep in aluminum trays containing natural soil at room temperature. Water was sprayed every day, and after 15 days, they were weighed [28]. The degradation of gelatin films was evaluated in terms of the percentage of weight lost using the following equation:

WL (%) = ((W_0_ − W_1_)/W_0_) × 100

WL: Gelatin film weight loss percentage;

W_0_: Initial weight of gelatin film;

W_1_: Final weight of film.

### 2.10. Statistical Analysis

All experiments were conducted in triplicate, and the data were analyzed by one-way ANOVA. Data were analyzed using SPSS version 23 (IBM, Armonk, NY, USA) with a significance level of *p* ≤ 0.05 (Tukey’s test).

## 3. Results and Discussion

### 3.1. Physicochemical Analysis of PP and FP

The peel is the inedible part of the prickly pear fruit due to the presence of spines. It is considered a by-product of the manufacturing process. There is a wide range of prickly pear varieties with differing physicochemical and nutritional compositions, making the peel a valuable source of compounds with high value-added potential [24]. Table 2 presents the chemical composition of the powdered peel. The measured moisture content was 3.66 ± 0.57%. This value is lower than those reported by other studies: Bourhia (2020) [29] found values of 10.80 ± 0.05% and 15.57 ± 0.02% for prickly pears from the Mediterranean, while Ettalibi (2018) [30] reported humidity around 20% for powder peel from the center of Morocco. Fresh pulp moisture was 79.63 ± 0.52%; previous reports have shown values between 86.00 and 89.05% in prickly pears from different harvest seasons [29,31]. Protein contents of PP and FP were 4.41 ± 0.06% and 0.44 ± 0.01%, respectively. Valero-Galván [32] reported 3.4 ± 0.2% for pulp and for peel 2.3 ± 0.0% of protein of different varieties of prickly pears from Chihuahua, Mexico. Crude fiber content was 9.41% for PP and 0.83 ± 0.02% for FP, which is higher than the value reported by Todaro [33] (5.32%) for Italian PP samples. Crude fiber in FP was 0.827 ± 0.02%; a similar result was obtained for Abou-Zaid [34], who reported 0.86% in red prickly pear.

Fat content in PP was 2.50 ± 0.73% and in FP 0.25 ± 0.02%. Previous studies have reported lower fat concentrations (0.32–1.69%) [11,35]. PP exhibited higher fat content than FP, likely due to the abundance of lipids in seeds and peel tissues. Ash content indicates the presence of minerals such as K, Ca, Mg, Na, Fe, and Cu, which are mainly concentrated in the pulp and peel of the prickly pear fruit. PP showed 11.96% of ash; these results are higher than Bellumori [36], who reported 11.0% of PP in prickly pear fruit from Sicily. The FP presented for ash 0.79%, which is lower than the reported value (2.6%) for El-Beltagi [27], who obtained the pulp from a mechanical press. PP presented 49.2% and FP 97.68% carbohydrate concentration. In FP, the increase in carbohydrates is due to the presence of monosaccharides, mainly fructose and glucose, and in peel can be present sucrose, stachyose, mannitol, arabinose, and sorbitol. Differences in the physicochemical composition of PP and FP can be attributed to plant type and origin, climate, and agronomic factors such as cultivation, fertilization, and irrigation practices [11].

### 3.2. Antioxidant Activity of PP and FP from Opuntia guerrana

The antioxidant and health-promoting properties of *Opuntia guerrana* are associated with the presence of bioactive compounds such as carotenoids, betaxanthins, betacyanins, flavonoids, and phenolic acids [37]. Table 3 shows the concentration of bioactive compounds such as phenols in powdered peel PP and fresh pulp FP, 3611.94 ± 22.39 mg EAG/mL/g and 1810.95 ± 15.02 mg EAG/mL/g, respectively, with significant differences (*p* ≤ 0.05). These results are higher than previous reports, where red and orange peel showed 1540 mg EAG/100 g and 136.90 mg EAG/100 g [38]. Flavonoids constitute approximately half of the total phenolic compounds and are largely responsible for the color of Opuntia fruits. In PP, the flavonoid concentration was 906.67 ± 8.10 mg EQ/g, and for FP 566.96 ± 2 7.21 mg EQ/g, showing significant differences (*p* ≤ 0.05). The *Opuntia* family presents a variety of colors that are related to the presence of bioactive compounds such as anthocyanins, carotenoids, and betalains. Betalains are nitrogenous pigments derived from a tyrosine base and produced from betalamic acid; they can be further categorized into betacyanin (purple-red color) and betaxanthins (yellow-orange color) [37]. In FP, betalin concentration was 139.05 ± 1.96 mg/g and PP 128.53 ± 0.34 mg/g, presenting significant differences (*p* ≤ 0.05) between them. The presence and concentration of bioactive compounds depend on factors such as Opuntia species, fruit part (peel or pulp), harvest season, storage conditions, ripening stage, and genetic characteristics [39]. Previous studies have shown a strong and positive correlation between the presence of bioactive compounds such as phenolics, flavonoids, and betalains and antioxidant activity because they interact with free radicals to prevent their oxidation [40]. In prickly pear fruits, bioactive compounds can be present in the peel and pulp; however, the concentration of these types of compounds depends directly on the method of extraction, affecting their functional capacity as scavengers of free radicals like ABTS and DPPH. DPPH radical inhibition by PP was 71.48 ± 2.68%, whereas FP showed 38.10 ± 1.50% inhibition. Peel from different varieties of prickly pear (red-purple) obtained the highest percentage of inhibition of the DPPH radical [41]. For the ABTS radical, PP inhibited 98.19 ± 0.21% and FP 67.42 ± 0.35% of the radical. The higher inhibition observed in PP is attributed to its greater content of antioxidant constituents, particularly phenolic compounds, in the peel fraction.

### 3.3. Gelatin Films Physicochemical Properties

#### Fourier Transform Infrared Spectroscopy

**Figure 1:**
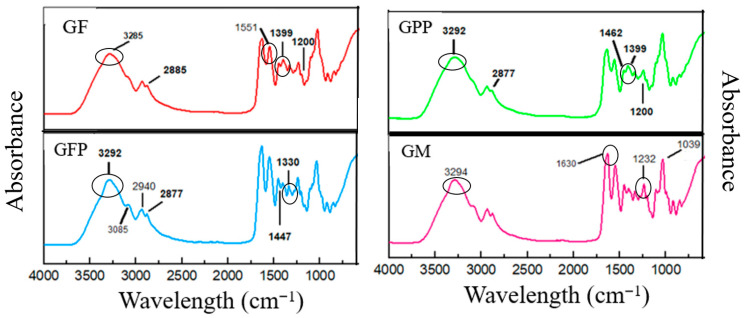
FTIR spectrum of gelatin films with PP and FP of *Opuntia guerrana*.

FTIR spectroscopy was employed to identify functional groups and their interactions within the biopolymer matrix through infrared transmission [1]. Figure 1 shows the spectrum of the different formulations of gelatin-based films with Opuntia compounds. All formulations presented characteristic bands of gelatin at 3294 cm^−1^ Amide A (stretching vibrations in N-H and O-H groups), 2940 cm^−1^ Amide B (C-H groups), 1625 cm^−1^ Amide I (C=O groups associated with COO within the protein structure), 1551 cm^−1^ Amide II (N-H bonds with vibrations of C=O groups), and 1232 cm^−1^ Amide III (vibrations of C-N and N-H groups) [40]. A peak between 1031 and 1039 cm^−1^, corresponding to glycerol OH groups, was observed in all formulations. Changes in the FTIR band intensity between 3294 and 3284 cm^−1^ in GPP, GFP, and GM formulations can be attributed to interactions between NH groups of gelatin and OH groups of phenolic compounds from *Opuntia guerrana*, forming hydrogen bonds with carbonyl groups. Also, a change was observed related to Amide I; in GM formulation the peak was present at 1639 cm^−1^, and for the control GF at 1630 cm^−1^, and this change can be due to the interaction of non-covalent bonds between the base matrix gelatin and the phenolic compounds of PP and FP. Amide II and Amide III bands in the control GF formulation were detected at 1559 and 1232 cm^−1^, respectively; in formulations containing PP and FP, these bands shifted to 1550 and 1249 cm^−1^, indicating that the compounds from *Opuntia guerrana* induced conformational changes and reduced gelatin–gelatin interactions in the composite films [42]. Other bands also observed at 3085 cm^−1^ may be related to the binding of vinylic H linked =C in GF. Bands related to C-H stretch were observed at 3000 and 2800 cm^−1^ in formulations GFP, GPP, and GM. The presence of bioactive compounds such as phenols is related to the band in the region 1390–1330 cm^−1^ (O-H deformation and C-O stretching); flavonoid bands are present in the region 1200 and 950 cm^−1^ (C-O stretching); the bands representative of the aromatic compounds are in the region 1462 cm^−1^; and the bands at 1399 cm^−1^ are related to simple aromatic ring and carboxyl stretching vibrations (hydroxybenzoic and hydroxycinnamic acids) in GPP and GM formulations. The signal at 1447 cm^−1^ represents the C=C aromatic rings present in the GFP formulation [43].

### 3.4. Gelatin-Based Films Optical Properties

Optical properties serve as indicators of biopolymer film quality, influencing product appearance and, consequently, consumer acceptability. In gelatin-based films with *Opuntia* compounds, the control presented 91.08 ± 1.03 of luminosity (L), and the films with *Opuntia guerrana* compounds showed the lowest luminosity (Table 4), showing significant differences (*p* ≤ 0.05) between formulations. The results are a* (negative: green, positive: red) and b* (negative: blue and positive: yellow) [44].

Similar results were exposed by Iahnke [45,46], where they obtained 24.91 of L in a gelatin base film with beet powder; also for Aparicio-Fernández [47], who added prickly pear peel powder to a carboxymethylcellulose matrix and obtained a value of 50.8 for L; and Espino-Manzano [14] showed a lower L value 44.6 when xoconostle extract was added to the gelatin base film. These results suggest that the addition of *Opuntia guerrana* compounds decreases luminosity due to pigments such as betalains and anthocyanins which are naturally present in prickly pears. Table 4 shows the results of a* (negative: green, positive: red) and b* (negative: blue, and positive: yellow). GM formulation showed the highest value in a* 41.45 ± 1.63, which indicates reddening color. Also, b* resulted in significant differences (*p* ≤ 0.05) in all formulations, including the control (−1.74 ± 0.23) to GPP (10.73 ± 1.31), indicating the rise in yellow color intensity. *Opuntia guerrana* is rich in betalains, which are glycosylated pigments that contribute to prickly pear fruit color. Betalains present with a red color due to the presence of betacyanin and yellow due to betaxanthins. The *Opuntia* pulp contains a higher proportion, hence the films GFP and GM present red color [48].

Opacity is an important parameter for evaluating gelatin-based films, as it reflects their resistance to photo-oxidation processes affecting food products [44]. When 2% of PP and the mix of PP and FP were added, there were significant differences (*p* ≤ 0.05), increasing the opacity in films. The addition of these prickly pear by-products increased the concentration of phenolic compounds, thereby affecting light dispersion [23]. The results obtained are similar to previous results, where it was found that by increasing the concentration of natural antioxidants from the peel or pulp from different fruits in gelatin-based films, the opacity value increased [49]. Optical properties of the different gelatin-based films were affected by the concentrations of PP and FP added, increasing red color and decreasing brightness.

### 3.5. Gelatin Films Barrier Properties

Water vapor permeability (WVP) is a critical parameter to understand the shelf life stability of food products, as water vapor penetration across packaging boundaries affects microbial growth, product texture, and functionality [3]. The control presented 5.81 × 10^−10^ g/m s Pa, GFP 6.15 × 10^−10^ g/m s Pa and GM 6.74 × 10^−10^ g/m s P with no significant differences (*p* > 0.05) between the formulations. However, GPP showed the highest WVP value of 7.84 × 10^−10^ g/m s Pa. The change in WVP value can be related to the presence of *Opuntia guerrana* compounds, causing also a loss of cohesion of the polymer network and changes in thickness which directly affects the WVP, causing an excessive presence of oxygen and generating lipid oxidation and microbial deterioration of the food during transportation or storage [40,50].

Formulations GF, GFP, and GM showed around 36% of humidity content, with no significant differences (*p* > 0.05), due to the *Opuntia* extract containing sugars that can act as a plasticizer in the film preparation reducing the interactions between polymer chains, which helps with water retention [42]. GPP formulation showed 31.8 ± 1.7% humidity content, presenting significant differences (*p* ≤ 0.05) compared to the control. The difference can be related to hydrophobic components present in the film that can form hydrophobic interactions with the biopolymer matrix and consequently give lower results in humidity content. Similar results were observed in reports where the presence of sugar, a hydrophilic compound, was added to the gelatin films [42].

Formulations showed 92.7 ± 1.6%, 92.3 ± 0.1%, 91.23 ± 1.60%, and 91.4 ± 1.6% for GF, GFP, GPP, and GM, respectively, with no significant differences (*p* > 0.05) in solubility parameter. In gelatin films, solubility is related to the presence of hydrophilic amino acids (lysine, serine, arginine, hydroxyproline, and aspartic and glutamic acids) and the phenolic compounds of *Opuntia guerrana* fruit that facilitate water-soluble components to leak into the composite films, resulting in an increase in the solubility [42,50]. When the percentage of solubility is high that may indicate low water resistance, facilitating disintegration and biodegradation, as well as in situations where the films can be consumed together with the product that is heated before consumption [23]. Table 5 shows significant differences (*p* ≤ 0.05) among GF (504 ± 2.9%) and GFP (309.40 ± 59.07%) with GPP (238.93 ± 12.40%) and GM (325.23 ± 17.17%) The presence of the pulp and peel from the prickly pear can decrease the interaction of water molecules, due to the increase in hydrophobicity coming from aromatic rings that react with the amino groups of the amino acids in the gelatin structure, and also are involved in cross-linking reactions, resulting in low availability to interact with water. When the swelling degree is high, the cross-linking will decrease because the gelatin chains will be short and less water molecules can be retained. Previous reports showed the same behavior as when adding powder from different fruits, ferulic acid, and tannic acid, the swelling index decreased in gelatin films [46].

### 3.6. Thermal Properties of Gelatin Films

**Figure 2:**
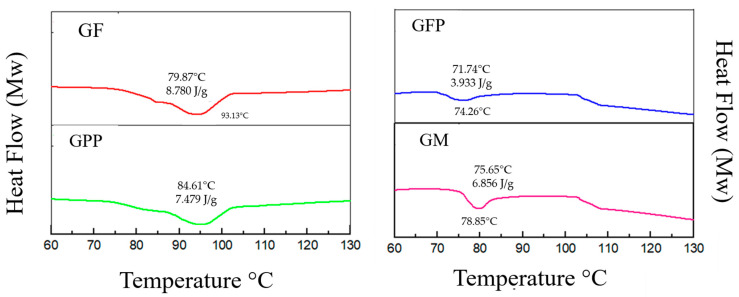
DSC of gelatin films with *Opuntia guerrana* peel and pulp.

Powdered peel and fresh pulp from prickly pears are a complex mixture of polyphenols, lipids, cellulose, minerals, pectin, and vitamins [6]. When they are added to gelatin films, they can affect the thermal properties, as we can see in Figure 2. The results showed that GPP and GM had a glass transition (Tg) of 84.61 °C and 79.87 °C, respectively, due to the peel, and the mix (pulp + peel) can act as a restrictive agent of gelatin molecules’ mobility, making an increase in Tg. For GFP formulation, Tg decreases to 71.74 °C; this can be related to the addition of fresh pulp, which can contain hydrophilic compounds such as sugars which act as a plasticizer by decreasing viscosity and causing structural relaxation which prevents protein–protein interactions [1]. Melting temperature results show that GFP was 74.26 °C, GPP 93.97 °C, and GM 93.13 °C. When powdered peel and fresh pulp were incorporated into gelatin films, they can form hydrophobic interactions between the prickly pear compounds and gelatin chains; also, the Tm can be related to the low volume used in film formulation [51].

### 3.7. Mechanical Properties of Gelatin Films

Film thickness is a parameter that impacts mechanical properties and can affect the quality and shelf life of food products [1]. An increase in solid content in the films could cause structural changes due to the loss of the gelatin network in the presence of PP and FP compounds. Formulations GPP and GM presented higher thickness values (Table 6), showing significant differences (*p* ≤ 0.05) with GF and GFP. Previous reports have observed the same change in thickness in gelatin-based films added with peel and pulp extract from different fruits, such as mango [21], beet root [46], and pineapple [25]. The thickness difference in edible films from biopolymers can be related to the source of the biopolymeric matrix, the film’s method of obtention, and the interactions between the components of the film [44].

The highest tensile strength (Ts) value was reported for GF (222.57 ± 6.12 MPa) and the lowest for GM (60.02 ± 2.28 MPa), showing significant differences (*p* ≤ 0.05) (Table 6). When powder residues and extracts from different natural sources (fruits and vegetables) are added in high concentrations into biopolymer matrixes as gelatin films, the tensile strength decreases due to several factors, like the formation of hydrogen and covalent bonds generated between antioxidant compounds (polyphenols) and amino and hydroxyl groups of the biopolymeric matrix, which weaken the protein–protein interactions responsible for the integrity of the biopolymer matrix [25,42]. Another factor that contributes to reducing flexibility is the presence of sugars in high amounts in the fresh pulp of the prickly pear fruit, which acts as a plasticizer, decreasing cohesion between the matrix and the fruit residue [52].

For elongation at break (EAB), the formulations GFP (44.2 ± 1.38%) and GM (43.10 ± 1.25%) showed a significant difference (*p* ≤ 0.05) with the control (GF, 18.33 ± 0.10%) and the film with powdered peel (GPP, 19.22 ± 0.5%). The presence of compounds from the prickly pear reduces intermolecular forces between gelatin polymer chains and consequently promotes film flexibility. Similar results were observed in gelatin films, where the incorporation of polymers (polysaccharides, chitin), natural extracts (boldo, teas, fruit and vegetable purees and peels, cinnamon, essential oils) achieves high elongation values [53]. Also, the source of gelatin can affect EAB, because gelatin from porcine presents a higher content of amino acids, which improve the viscoelastic properties, and a greater capacity to develop a stronger gel structure, which favors a higher EAB [54].

Low values of Young’s modulus indicate that the film is more flexible, and high values correspond to a rigid film [55]. Formulations GFP 0.157 ± 0.01 MPa, GPP 0.155 ± 0.06 MPa, and GM 0.054 ± 0.00 MPa showed significant differences (*p* ≤ 0.05) compared to the control. When a high concentration of bioactive compounds, such as phenolics and flavonoids from *Opuntia guerrana* pulp and peel, is incorporated into the biopolymer matrix, Young’s modulus decreases. Similar results were observed with leaf extract from *Ficus carica* and quercetin in different concentrations in gelatin-based films [22].

### 3.8. Functional Properties of Gelatin Films

When a molecule can prevent or delay an oxidation reaction, it is considered an antioxidant [56]. The incorporation of antioxidants into gelatin films will help to develop its functionality. Phenols, flavonoids, and betalain present in prickly pear fruit have been reported to have an excellent antioxidant activity [20]. GGP formulation presented the highest bioactive compound concentration, with 1304.98 ± 23.14 mg EAG/g, 1289.02 ± 12.51 mg EQ/g, and 119.89 ± 1.46 mg/g for phenols, flavonoids, and betalain, respectively (Table 7). The formulations GFP, GPP, and GM presented significant differences (*p* ≤ 0.05). Other researchers reported lower phenol content when they added compounds from different varieties of prickly pear and fruits such as *Opuntia oligacanta*, *Opuntia ficus-indica L*., and mango [14,21,47]. The presence of phenols, flavonoids, and betalain will depend on the prickly pear fruit variety, the method of extraction of its pulp, time, and the temperature of the powdered peel obtention [42]. The highest inhibition for DPPH radical was shown in formulation GM (42.72 ± 1.56%) and for the ABTS radical as well (98 ± 0.14%). The inhibition of both radicals increased when the *Opuntia guerrana* compounds are added to the gelatin matrix, showing significant differences (*p* ≤ 0.05) among formulations GFP, GPP, and GM (Table 7). The results are similar to those reported in other works, where it was confirmed that adding or incorporating natural compounds increases the antioxidant properties in the films [47,52]. Also, antioxidant activity is not specific to bioactive compounds from fruit sources, and proteins such as gelatin present bioactive function by radical inhibition due to the presence of peptides [56].

### 3.9. Biodegradability of Gelatin Films

**Figure 3:**
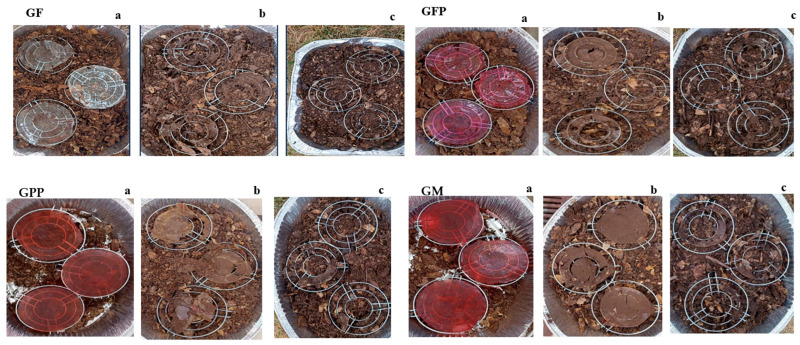
Biodegradability of gelatin films with tuna compounds (a: day 0; b: day 7; c: day 15).

Biodegradability can be defined as the change that occurs when the biopolymeric material structure is fractionated and the film is converted into carbon dioxide, water, methane, inorganic materials, and biomass by the action of microflora present in the soil. Gelatin films were buried in organic soil with the intention of reproducing natural environment biodegradation conditions for 15 days. GF and GFP showed higher biodegradability on day 7; GPP and GM presented a low biodegradability on the same days of analysis (Figure 3). At the end of the analysis, GF and GFP had lost around 96 ± 6.2% of their initial weight, and GPP 93 ± 5.9% and GM 77.33 ± 1.84%, respectively. The difference in weight loss of the films can be related to the presence of hydroxyl groups of hydroxyproline, which are available to bind to water molecules by hydrogen bonds, making gelatin-based films more susceptible to microbial attack due to their high moisture content. However, the presence of bioactive compounds from *Opuntia guerrana* added to gelatin films can interfere with microbial growth in the soil limiting the biodegradability of the films. Other authors, such as Iahnke [46], reported that adding beet husk residue decreased the biodegradability in gelatin-based films. Biodegradability is a very important factor to evaluate in biopolymer films, as the complete disintegration of these materials indicates full biodegradability, and they can be considered biodegradable and environmentally friendly packaging [25].

## 4. Conclusions

Bioactive compounds from *Opuntia guerrana* can be successfully incorporated into gelatin-based films, significantly modifying their physicochemical and functional properties. The incorporation of peel and fresh pulp increased antioxidant activity and the degree of biodegradability, resulting in more flexible films, with modified barriers and thermal properties. The color of the films is related to the concentration of bioactive compounds, obtaining reddish gelatin films upon addition of the pulp and peel. These findings support the potential of *Opuntia guerrana* residues as sustainable sources of natural antioxidants for developing biodegradable and functional food packaging materials

## Figures and Tables

**Table 1:** Gelatin edible films formulations with different prickly pear compounds.

Formulations	Gelatin (*w*/*w*)	Solvent (*v*/*v*)	Glycerol (*w*/*v*)	PP (*w*/*v*)	FP (*w*/*v*)
GF	2%	180 mL	1%	-------	-------
GFP	2%	180 mL	1%	-------	2%
GPP	2%	180 mL	1%	2%	-------
GM	2%	180 mL	1%	2%	1%

GF: gelatin film; GFP: gelatin + fresh pulp; GPP: gelatin powdered peel; GM: gelatin mix (PP and FP).

**Table 2:** Chemical composition of powdered peel and fresh pulp obtained from *Opuntia guerrana*.

Parameter	PP (%*w*/*w*)	FP (%*w*/*w*)
Humidity	3.66 ± 0.57	79.63 ± 0.52
Protein	4.41 ± 0.06	0.440 ± 0.01
Fat	2.50 ± 0.73	0.251 ± 0.02
Ash	11.96 ± 0.15	0.793 ± 0.00
Fiber	9.41 ± 1.53	0.827 ± 0.02
Carbohydrates	49.2	97.68

**Table 3:** Bioactive compounds and antioxidant activity in powder peel and fresh pulp from *Opuntia guerrana*.

Sample	Phenols (mg EAG/mL/g)	Flavonoids (mg EQ/g)	Betalains (mg/g)	DPPH Inhibition (%)	ABTS Inhibition (%)
PP	3611.94 ± 22.39	906.67 ± 8.10	128.53 ± 0.34	72.82 ± 2.86	98.19 ± 0.21
FP	1810.95 ± 15.02	566.96 ± 27.21	139.05 ± 1.96	38.10 ± 1.50	67.42 ± 0.35

**Table 4:** Optical properties of gelatin-based films with *Opuntia guerrana* compounds.

Formulation	L	a*	b*	Opacity	
GF	91.08 ± 1.03 ^a^	−0.31 ± 0. 14 ^a^	−1.74 ± 0.23 ^b^	0.98 ± 0.06 ^a^	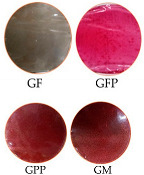
GFP	67.59 ± 2.16 ^b^	33.53 ± 2.34 ^b^	−15.39 ± 1.03 ^a^	1.06 ± 0.00 ^a^	
GPP	46.26 ± 1.14 ^c^	39.53 ± 2.28 ^c^	10.73 ± 1.31 ^d^	2.31 ± 0.01 ^b^	
GM	43.52 ± 1.80 ^d^	41.45 ± 1.63 ^d^	8.82 ± 1.46 ^c^	2.64 ± 0.09 ^c^	

**Table 5:** Barrier properties of gelatin films with *Opuntia guerrana* compounds.

Formulation	WVP (g/m s Pa)	Humidity (%*w*/*w*)	Solubility (%*w*/*w*)	Swelling Degree (%*w*/*w*)
GF	5.81 × 10^−10^ ± 2.27 × 10^−8 b^	31.82 ± 1.76 ^a^	92.65 ± 1.63 ^a^	237.19 ± 18.05 ^a^
GFP	6.15 × 10^−10^ ± 1.28 × 10^−8 b^	36.71 ± 1.31 ^a^	92.34 ± 0.12 ^a^	309.40 ± 59.07 ^a^
GPP	7.84 × 10^−10^ ± 1.05 × 10^−9 ab^	36.44 ± 1.82 ^b^	91.23 ± 1.62 ^b^	238.93 ± 12.40 ^b^
GM	6.74 × 10^−10^ ± 5.1 × 10^−9 b^	36.00 ± 0.67 ^b^	89.69 ± 0.60 ^a^	325.23 ± 17.17 ^c^

**Table 6:** Mechanical properties of gelatin films.

Formulation	Thickness (mm)	TS (MPa)	EAB %	YM (MPa)
GF	0.098 ± 0.01 ^c^	222.57 ± 6.12 ^a^	33.50 ± 1.41 ^b^	0.328 ± 0.06 ^a^
GFP	0.121 ± 0.01 ^b^	163.20 ± 3.14 ^b^	42.20 ± 1.38 ^a^	0.157 ± 0.01 ^b^
GPP	0.278 ± 0.01 ^a^	57.32 ± 3.82 ^c^	18.33 ± 0.10 ^c^	0.155 ± 0.06 ^b^
GM	0.275 ± 0.01 ^a^	60.02 ± 2.28 ^c^	43.10 ± 1.25 ^a^	0.054 ± 0.02 ^c^

**Table 7:** Concentration of phenols, flavonoids, and betalain content in gelatin films.

Formulation	Fenols (mg EAG/g)	Flavonoids (mg EQ/g)	Betalain (mg/g)	ABTS Inhibition %	DPPH Inhibition %
GF	------------	------------	-----------	42.31 ± 0.82 ^d^	12.30 ± 1.57 ^c^
GFP	625.87 ± 3.76 ^c^	175.29 ± 5.88 ^c^	20.30 ± 0.34 ^c^	65.28 ± 0.14 ^c^	15.42 ± 2.61 ^b^
GPP	1304.98 ± 23.14 ^a^	1289.02 ± 12.51 ^a^	119.89 ± 1.46 ^a^	98.24 ± 0.08 ^b^	38.50 ± 2.11 ^a^
GM	1144 ± 31.34 ^b^	1159.61 ± 21.33 ^b^	98.68 ± 0.64 ^b^	98.00 ± 0.14 ^a^	42.72 ± 1.56 ^a^

## Data Availability

The data presented in this study are available on request from the corresponding authors.

## Abbreviations

The following abbreviations are used in this manuscript:

PP	Powder peel
FP	Fresh pulp
GF	Gelatin film
GFP	Gelatin film + fresh pulp
GPP	Gelatin film + powder peel
GM	Gelatin film + mix (PP and FP)
